# Skeletal muscle effects of two different 10‐week exercise regimens, voluntary wheel running, and forced treadmill running, in mice: A pilot study

**DOI:** 10.14814/phy2.14609

**Published:** 2020-10-29

**Authors:** Angelika Schmitt, Pascal Herzog, Franziska Röchner, Anne‐Lena Brändle, Annunziata Fragasso, Barbara Munz

**Affiliations:** ^1^ Department of Sports Medicine Medical Clinic University Hospital Tübingen Tübingen Germany; ^2^ Interfaculty Research Institute for Sport and Physical Activity Eberhard Karls University of Tübingen Tübingen Germany

**Keywords:** forced treadmill running, mice, skeletal muscle, voluntary wheel running

## Abstract

Physical activity and exercise induce a complex pattern of adaptation reactions in a broad variety of tissues and organs, particularly the cardiovascular and the musculoskeletal systems. The underlying mechanisms, however, specifically the molecular changes that occur in response to training, are still incompletely understood. Animal models help to systematically elucidate the mechanisms of exercise adaptation. With regard to endurance‐based running exercise in mice, two basic regimens have been established: forced treadmill running (FTR), usually consisting of several sessions per week, and voluntary wheel running (VWR). However, the effects of these two programs on skeletal muscle molecular adaptation patterns have never been directly compared. To address this issue, in a pilot study, we analyzed the effects of two ten‐week training regimens in juvenile, male, C57BL/6 mice: moderate‐intensity forced treadmill running three‐times‐a‐week, employing a protocol that has been widely used in similar studies before, and voluntary wheel running. Our data suggest that there are similarities, but also characteristic differences in the molecular responses of different skeletal muscle species to the two training regimens. In particular, we found that VWR induces a significant fiber type shift toward more type IIX fibers in the slow, oxidative soleus muscle (*p* = .0053), but not in the other three muscles analyzed. In addition, while training‐induced expression patterns of the two metabolic markers *Ppargc1a, encoding* Pgc‐1α (peroxisome proliferator‐activated receptor gamma coactivator 1‐alpha) and *Nr4a3* (nuclear receptor subfamily 4 group A member 3) were roughly similar, downregulation of the *Mstn* (myostatin) gene and the “atrogene” *Fbox32* could only be observed in response to VWR in specific muscles, such as in the gastrocnemius (*p* = .0015 for *Mstn*) and in the tibialis anterior (*p* = .0053 for *Fbox32*) muscles, suggesting that molecular adaptation reactions to the two training regimens show distinct characteristics.

## Graphical abstract

Forced treadmill and voluntary wheel running have differential effects on mouse skeletal muscle, specifically with regard to expression of exercise‐associated genes and miRNAs. In addition, effects differ significantly between individual muscle types.

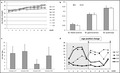

## 1 INTRODUCTION

It is well known that physical activity is an important preventive, therapeutic, and rehabilitative tool with regard to most acute and chronic diseases. However, the molecular mechanisms that modulate exercise adaptation are still incompletely understood. In skeletal muscle, basic signals that initiate adaptation might be (a) mechanical signals, such as stretching, muscle damage or increased blood flow, (b) enhanced activity at the neuro‐muscular junction and oscillating calcium concentrations associated with muscle activity, (c) energy depletion, such as a decreasing ATP/ADP ratio, and (d) systemic factors, such as hormones, growth factors and cytokines (for review, see Egan et al., 2016; Egan and Zierath, 2013; Hamilton and Booth, 2000; Wackerhage and Woods, 2002). These signals then activate specific signal transduction cascades, which eventually regulate expression of adaptation‐relevant genes.

To develop and optimize preventive, therapeutic, and rehabilitative training regimens in clinical practice, a deeper understanding of the mechanisms involved in exercise adaptation is important. Animal, particularly rodent models help to elucidate these mechanisms in a standardized manner. With regard to endurance exercise training, the two main regimens are moderate‐intensity forced treadmill running (FTR), usually on 3–5 days per week for 20–90 min, at speeds of about 10–20 m/min and 0–15° incline, or voluntary wheel running (VWR), for which animals’ cages are equipped with running wheels and the distances run are documented (Goh and Ladiges, 2015; Manzanares et al., 2018). While in general, skeletal muscle adaptation reactions can be observed with both protocols (for review, see Guo et al., 2020), to our knowledge, their specific effects in similar cohorts of mice have only been directly compared in one recent study (Kim et al., 2020), with a particular focus on body composition, metabolism and muscle force. Consequently, little is known on the differential effects of the two training regimens on skeletal muscle molecular biology, specifically effects on gene expression and miRNA concentrations.

Each skeletal muscle consists of different types of muscle fibers, with characteristic metabolic and functional properties: So‐called type 1 or “red” fibers contract slowly and are characterized by an “oxidative” metabolism, whereas type 2 fibers contract faster and show a predominantly “glycolytic” metabolism. While a lot of intermediate forms and also “hybrid” fibers exist, metabolic and functional adaptation to a particular type of exercise can occur by the so‐called “fiber type switching,” which means that endurance training favors shifts toward “slower” and resistance training toward “faster” isotypes. These adaptation reactions are paralleled by changes in the expression of genes encoding characteristic fiber type‐specific myosins: While resistance exercise enhances expression of “fast” myosin heavy chain (MyH) genes, endurance training induces expression of genes encoding “slower” MyH isoforms (Medler, 2019). Similarly, the gene encoding the sarcomere component alpha actinin 3 (*Actn3*) is a marker of “fast” skeletal muscle fibers and adaptation toward a more glycolytic metabolism (Ogura et al., 2008).

Correspondingly, depending on the type of exercise, training also induces characteristic metabolic adaptations in skeletal muscle cells, reflected by altered expression patterns of genes encoding mitochondrial and metabolism‐associated factors, such as Pgc‐1α (Peroxisome proliferator‐activated receptor gamma coactivator 1‐alpha), a regulator of mitochondrial biogenesis, Ucp3 (uncoupling protein 3), a protein that uncouples proton flux and ATP synthesis in mitochondria, or Cox4 (cytochrome c oxidase subunit 4), a component of the mitochondrial respiratory chain. Specifically, whereas the *Ppargc1a* gene, encoding Pgc‐1 α, as well as *Cox4,* have been shown to be upregulated in response to endurance exercise in a variety of contexts (Burgomaster et al., 2007; Handschin and Spiegelman, 2008; Lira et al., 2010; Short et al., 2003; Southern et al., 2017; Sylviana et al., 2019), *Ucp3* expression appears to be upregulated by acute exercise, but downregulated by endurance exercise training. However, this issue has been controversially discussed (Hesselink et al., 2003; Schrauwen and Hesselink, 2003; and references therein).

Physical exercise, specifically resistance or mixed‐type training, can also repress muscle atrophy and induce hypertrophy, associated with characteristic changes in gene expression. Important markers in this context are the *Mstn* gene, which encodes the transforming growth factor‐beta (TGF‐α) family member myostatin, the *Murf1* gene, which encodes muscle ring finger protein 1 (Murf1 or Trim63), or *Fbox32,* encoding atrogin‐1 (Fbox32, MAFbx). All three genes have been shown to be repressed by exercise and induced in situations of skeletal muscle atrophy (Murton et al., 2008; Lightfoot and Cooper, 2016).

A major group of genes that are tightly regulated in response to physical activity are inflammation‐ and anti‐inflammation‐associated genes: In general, acute exercise induces inflammation, whereas chronic training has been shown to reduce both systemic and skeletal muscle inflammation (for review, see Beiter et al., 2015). Particularly the IL‐6 (interleukin‐6) pathway has gained a lot of attention: Systemic IL‐6 concentrations are upregulated in response to acute exercise, whereas both acute and long‐term, chronic endurance exercise have been shown to induce expression of the *Il6r* (interleukin 6 receptor) gene in skeletal muscle tissue (Beiter et al., 2015; Belizário et al., 2016; Keller et al., 2005). In parallel, genes related to anti‐inflammation, such as *Zfp36* (zinc finger protein 36) and *Ho1* (heme oxygenase 1) have been shown to be upregulated in response to acute and/or chronic exercise, respectively (Beiter et al., 2015;; Essig et al., 1997; Islam et al., 2020).

Finally, recent research suggests that exercise also induces characteristic alterations in the expression of genes encoding small regulatory RNAs (micro RNAs, miRNAs) and genes associated with the miRNA biogenesis pathway (for review, see Kirby and McCarthy, 2013; Meurer et al., 2016; Russell and Lamon, 2015; Silva et al., 2017; Silva et al., 2020; Sjögren et al., 2018; Ultimo et al., 2018; Widmann et al., 2019). In this context, specifically muscle‐specific and muscle‐enriched miRNAs, so‐called myomiRNAs, are interesting (for review, see Horak et al., 2016).

Given the diverse effects of different types of exercise on the murine body, specifically on skeletal muscle, it is important to better characterize and compare the effects of the two major training regimens, VWR and FTR, in different muscle types. For the pilot study described here, we subjected age‐ and sex‐standardized cohorts of inbred C57BL/6 mice to a 10‐week VWR training regimen. At the end of the training period, the effects on skeletal muscle gene expression at the mRNA and miRNA levels were analyzed. Furthermore, skeletal muscle tissue from two previous 10‐week FTR experiments (carried out as described in Schmitt et al., 2018) was analyzed in parallel, and, after normalization to the respective sedentary control groups, mRNA and miRNA expression data were compared. The results suggest that VWR and FTR exert characteristic and unique effects on skeletal muscle adaptation reactions, which might be due to different training workloads, but also the different training modes themselves, and that the effects were highly dependent on the respective muscle type.

## 2 MATERIALS AND METHODS

### 2.1 Animals

Male C57BL/6 (C57BL/6NCrl *H‐2^b^*) mice were purchased from Charles River, Sulzfeld, Germany, at 6–7 weeks of age. They were housed and fed according to federal guidelines. Animals were regularly inspected by a veterinarian. Their body weight was recorded and documented weekly.

### 2.2 Exercise

For voluntary wheel running (VWR), a group of eight mice was randomly divided into two *n* = 4 subgroups (sed = sedentary and ex = exercised). They were initially housed in groups of four and separated from each other six days before the start of the experiments due to repeated fighting or other types of aggressive behavior. All animals started wheel running at 8–9 weeks of age. For this purpose, they were housed in individual cages equipped with a plastic running wheel (PLEXX B.V., Elst, The Netherlands), connected to a standard bicycle speedometer (CM4.11, CicloSport, Gräfelfing, Germany). The total distance run was recorded and documented daily. The 10‐week FTR running protocol has already been described in Schmitt et al., 2018. Briefly, animals ran for an hour on Mondays, Wednesdays, and Fridays every week, with gradually increasing speed and incline, until they reached a final speed of 14m/min and 15° incline at the end of the fifth week. For the study described here, four randomly chosen mice from the two groups (sed and ex) were analyzed. With the exception of the soleus muscle (S) qPCR analyses, for which tissue from a different FTR experiment was used, all samples were from the same mice out of the same experiment (*n* = 8, with sed: *n* = 4 and ex: *n* = 4).

### 2.3 Isolation of muscle tissue

FTR animals were sacrificed two days after the last training session. Skeletal muscle tissue of all mice was dissected and subsequently either embedded in Tissue Tek® OCT (VWR), frozen in melting isopentane (Roth, Karlsruhe, Germany) and stored at −80°C until sectioning, or weighed and transferred into RNAlater® (Thermo Fisher Scientific, Waltham, MA, USA).

### 2.4 Fiber type quantification

For fiber type analysis, isolated skeletal muscles embedded in Tissue Tek® OCT were cross‐sectioned to obtain cryosections, which were subsequently subjected to fiber type analysis. For this purpose, mATPase staining after acidic preincubation (pH 4.5) was performed as previously described (Brooke and Kaiser, 1969; Chung, 2012; Kalmar et al., 2012; Muscle Physiology Laboratory, 2000; Ogilvie and Feeback, 1990). This method allows discrimination between type 1 fibers, which appear dark brown, type 2A fibers (very bright), and type 2X/2B fibers (intermediate). Briefly, cross sections (S: 10 µm, gastrocnemius muscle (G), tibialis anterior muscle (T), and quadriceps muscle (Q): 18 µm) were air‐dried at room temperature (RT) and pre‐incubated at pH 4.5. After washing with a Tris‐based washing buffer, sections were incubated in ATP solution (pH 9.4) for 25 min, then shortly rinsed with a 1% potassium chloride solution, washed with distilled water, and subsequently incubated in a 1% cobalt chloride solution. Sections were then washed with distilled water and incubated in a 1% ammonium sulfide solution. After three short washes in distilled water, sections were embedded in a water‐based mounting medium. For statistical analyses, five sections of each muscle type were photographically documented at 10‐fold magnification (Wilovert Standard, Hund Wetzlar, uEye IDS Obersulm). For G, Q, and TA, individual images were assembled into composite panoramic images and matched to low‐magnification survey images. Subsequently, all differentially stained skeletal muscle fibers within the entire muscle cross‐section were counted and statistically analyzed using *t* test. Fiber counts and fiber type percentages data are reported as group means ± *SD* (sed: *n* = 4; ex: *n* = 4).

### 2.5 Total and miRNA extraction

Total and miRNA from frozen muscle specimens of G, T, and S were isolated using the miRNeasy isolation kit from Qiagen (Hilden, Germany). In short, 2 to 3 mg of skeletal muscle tissue was homogenized in Qiazol® (Qiagen, Hilden, Germany) using the Beadbug homogenizer with Precellys® zirconium beads kit (VWR, Germany) for three times for 30 s, with cooling on ice between homogenization steps. The homogenized tissue was transferred to new vials, vortexed for 60 s, transferred to a QIAshredder column, and centrifuged at RT. After incubation at room temperature for 10 min, chloroform was added and the sample was vortexed for 15 s. The solution was again incubated at room temperature and centrifuged at 4°C for 15 min. Then, the upper aqueous phase containing the RNA and miRNA was transferred to a new vial, 1.5 volumes of ethanol were added, and the solution was transferred to a miRNeasy column for purification. After several washing steps with buffers included in the kit, and two additional washing steps with 70% ethanol, the RNA/miRNA was eluted from the column. RNA quantity and purity (260/280 ratio) were assessed using a BioPhotometer (Eppendorf AG, Hamburg, Germany).

### 2.6 Semi‐quantitative RT‐PCR (qPCR)

To analyze the expression of genes, 500 ng of total RNA per sample was reverse‐transcribed and analyzed by semi‐quantitative real‐time PCR (qPCR) as described in Schmitt et al., 2018. For miRNA analysis, 400 ng of total RNA/miRNA were used for reverse transcription using the miScriptII Kit (Qiagen, Hilden, Germany) in combination with HiSpec buffer in a total volume of 20 µl. The cDNA was diluted and employed in qPCR analyses using the miScript SYBR Green kit from Qiagen (Hilden, Germany) according to the manufacturer's instructions. For all experiments, melting curve analysis was carried out to confirm that a single transcript was produced. To calculate qPCR relative gene expression, the comparative CT (2^−ΔΔ^ *^C^*_T_) method was employed. Expression was normalized to *Gapdh, Hprt, Tbp,* and *Rps12* or housekeeping genes for miRNA analysis *SNORD95*, *SNORD96A,* and *RNU6‐2*. Primer sequences are listed in Tables 1 and 2. The following primers were purchased from Qiagen (Hilden, Germany): ZFP36/Tis11 (Mm_Zfp36_2_SG, QT01060962), and housekeeping genes for miRNA analysis Hs_SNORD95_11 (MS00033726), Hs_SNORD96A_11 (MS00033733), and Hs_RNU6‐2_11 (MS00033740).

**Table 1:** Gene‐specific Primers

Gene name	Forward primer (5´‐>3´)	Reverse primer (5´‐>3´)
Gapdh	TGTGTCCGTCGTGGATCTGA	TTGCTGTTGAAGTCGCAGGAG
Hprt	AGTACAGCCCCAAAATGGTTAAG	CACAAACGTGATTCAAATCCCTG
Tbp	AAGAGAGCCACGGACAACTGC	CTTCACATCACAGCTCCCCAC
Rps12	CTCATCCACGATGGCCTAGC	AGTGCCTCCACCAGCTTGAC
Cox4	CGCTCGTTCTGATTTGGGAG	GGCCTTCATGTCCAGCATTC
Nr4a3	AGATACCCTCCAGATATGCCCT	TGGTCAGCTTGGTGTAGTCG
Myh1	AAGGAGCAGGACACCAGCGCCCA	ATCTCTTTGGTCACTTTCCTGCT
Myh2	GCTTCAAGTTTGGACCCACG	ACTTCCGGAGGTAAGGAGCA
Myh7	GCTGGAAGATGAGTGCTCAGAG	TCCAAACCAGCCATCTCCTCT
Il6r	CTGCCCACATTCCTGGTAGC	TGGAGGAGAGGTCGTCTTGC
Ho1	AGGCTAAGACCGCCTTCCTG	AGCAGGCCTCTGACGAAGTG
Ppargc1α	GCTCATTGTTGTACTGGTTGGATATG	CGTAGGCCCAGGTACGACAG
Ucp3	AACCCAGGGGCTCAGAGCGT	GTCCGCTCCCTTGGGGGTGT
Actn3	CCCTCAGTTCGCAGGACATC	CCAGCTCCTCCTGCAGTGTC
Mstn	AACCTTCCCAGGACCAGGAG	TCGCAGTCAAGCCCAAAGTC
Murf1	GCAGCTCATCAAGAGCATTGT	CCAAAGTCAATGGCCCTCAA
Fbox32	GTGAGGACCGGCTACTGTGG	CAATCCAGCTGCCCTTTGTC
Xpo5	CCGTGCACGAATGAGCTTTT	AGGGGTTACGGAAGATGGGA
DGCR8	GGCGCCACAGGTGGAA	TACACACTGGCGGCTTA
Dicer	CTGAGCTTAGGAGATCCGAGG	CTTCCACGGTGACTCTGACC
Drosha	TCTCTGTAGAGACTGTGAATCCTG	GCTACATCTTCCGCTCACGA

**Table 2:** miRNA Primers

miRNA‐ID	miRBase accession number	Primer sequence (5’−3’)	bp primer
mmu‐miR‐494‐3p	MIMAT0003182	TGAAACATACACGGGAAACCTC	22 bp
mmu‐miR‐107‐3p	MIMAT0000647	AGCAGCATTGTACAGGGCTATCA	23 bp
mmu‐miR‐133a‐3p	MIMAT0000145	TTTGGTCCCCTTCAACCAGCTG	22 bp
mmu‐miR‐29a‐3p	MIMAT0000535	TAGCACCATCTGAAATCGGTTA	22 bp
mmu‐miR‐20a‐5p	MIMAT0000529	TAAAGTGCTTATAGTGCAGGTAG	23 bp
mmu‐miR‐20b‐5p	MIMAT0003187	CAAAGTGCTCATAGTGCAGGTAG	23 bp
mmu‐miR‐206‐3p	MIMAT0000239	TGGAATGTAAGGAAGTGTGTGG	22 bp
mmu‐miR‐1a‐3p	MIMAT0000647	TGGAATGTAAAGAAGTATGTATAAAA	26 bp

### 2.7 Statistical analysis

Statistical analysis was carried out using JMP software (Version 11; SAS Institute, Cary, NC, USA). The significance of fold changes between ex (VWR and FTR) and sed groups was tested with unpaired Student's *t* test. To test for differences between all four groups, one‐way ANOVA was performed. Data were considered significant with p‐values of less than 0.05 (*), less than 0.01 (**), or less than 0.001 (***). Data are presented as means ± *SD*.

## 3 RESULTS

Before comparing the molecular effects of the two training regimens, we first analyzed physiological data, particularly weight gain throughout the experiment, weight gain of individual muscles, distances run, and fiber type distribution, in the *de novo* VWR experiment.

### 3.1 Running distances and weight gain in VWR

**Figure 1:**
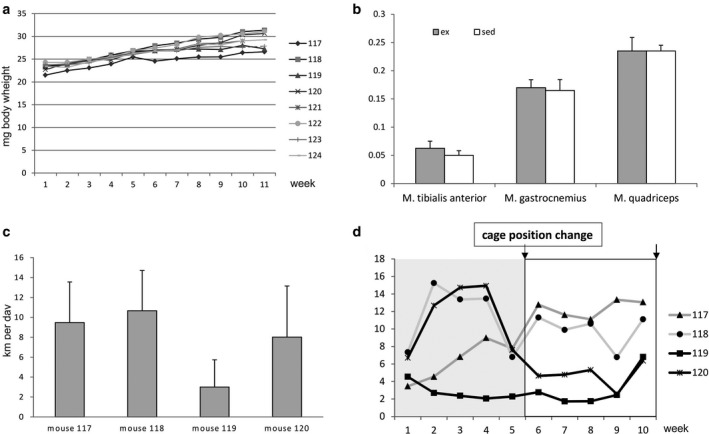
Physiological adaptation of animals to VWR. (a) Body weight of all mice (#117‐#120: ex: #121‐#124: sed) throughout the experiment is displayed. (b) Weight of individual muscles in the sed and in the ex group relative to body weight as indicated. (c) Average distances run by the four ex mice throughout the experiment. (D) Running distances for individual mice (weekly averages). The line marks the time point when cages were switched from the “higher” to the “lower” rack (#117 and #118) and *vice versa* (#119 and #120).

VWR was well tolerated by all animals and macroscopically, exercising animals were indistinguishable from sedentary controls. Correspondingly, weight gain was not significantly different between sed and ex mice (Figure 1a). Interestingly, ex mice showed a non‐significant trend toward increased weight of G and particularly T (Figure 1b). Animals of the ex group voluntarily ran 7.78 km (± 3.37 km) per day on average, with large inter‐ and intra‐individual variations (Figure 1c). Due to the impression that cage position in the rack influenced running activity, cages were swapped after five weeks: Cages #117 and #119 were transferred from a top to a bottom shelf, and cages #118 and #120 *vice versa*. This relocation had differential effects on animal running behavior, ranging from (further) increases after transfer to the bottom (#117) and decreases after transfer to the top (#118, #120), to no effect (#119) (Figure 1d).

### 3.2 Fiber type shifts in response to VWR

**Figure 2:**
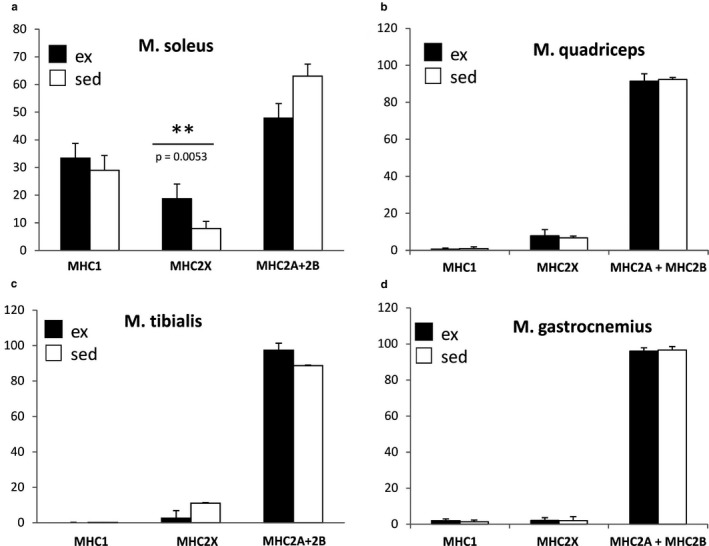
Percentages of individual fiber types in S, Q, T, and G as assessed by mATPase staining. Individual fiber types were counted on five sections per muscle and animal. Results are displayed as percentages of fibers of a specific type with regard to total fiber count.

Fiber type distribution in muscle cross‐sectional cryosections was assessed using mATPase staining. As shown in Figure 2, we could particularly detect increased percentages of type 2X fibers and decreased amounts of type 2A/2B fibers in the slow, “oxidative” S. Since the latter does not contain type 2B fibers, despite the fact that mATPase staining does not allow discrimination between 2A and 2B fibers, this result is specific for the former. Analysis of the fast, glycolytic T showed the opposite pattern (although discrimination between type 2A and 2B fibers was not possible here), whereas there were hardly any changes with regard to fiber type composition in the fast muscles Q and G.

### 3.3 mRNA and miRNA expression profiles in response to VWR and FTR

Skeletal muscle adaptation to VWR was analyzed in G, T, and S at the mRNA and miRNA levels. In addition, muscle tissue from two previous 10‐week FTR experiments with *n* = 16 mice of the same strain, sex, and age, was analyzed in parallel, in order to compare the effects of VWR and FTR. In these experiments, as described in Schmitt et al., 2018, mice were run on a treadmill three times a week for 60 min each, with gradually increasing speed and incline, until animals ran at a final speed of 14 m/min and 15° inline from the sixth week onwards. Expression levels (fold changes) of genes (or concentrations of miRNAs) of interest were first evaluated in comparison to the respective sedentary control groups and analyzed for significant differences. Subsequently, control groups were normalized to each other and the effects of the two training regimens were directly compared and again analyzed for significant differences. In the following, the results of these analyses are described.

#### 3.3.1 Genes encoding sarcomere components and sarcomere‐associated proteins

**Figure 3:**
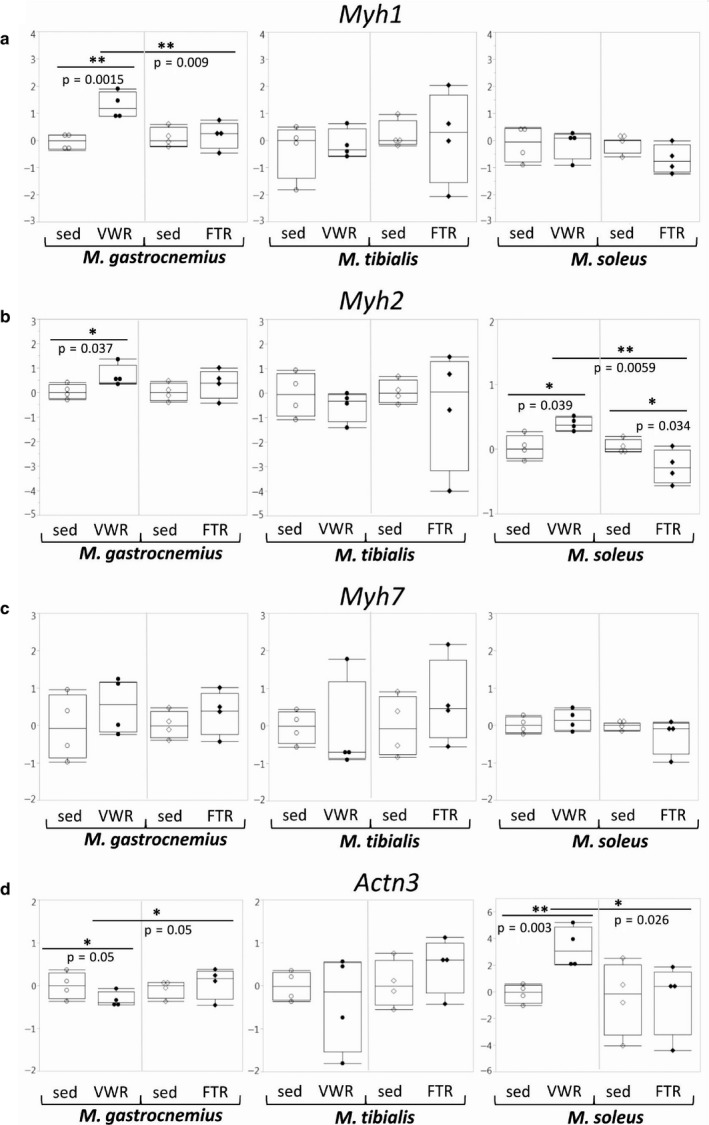
Expression of genes encoding Myh isoforms and Actn3. Expression of the genes encoding Myh1, Myh2, Myh7, and Actn3 was assessed by qPCR as indicated.

In the FTR group, there were no significant alterations with regard to expression of *Myh1* (encoding type 2X myosin heavy chain), *Myh2* (encoding type 2A myosin heavy chain), and *Myh7* (encoding type 1 myosin heavy chain) genes, except in S, where we detected a significant decrease in *Myh2* expression. In contrast, in the VWR group, we observed a highly significant induction of *Myh1* in G, as well as a significant induction of *Myh2* in both G and S. When comparing the effects of the two training regimens, higher levels of *Myh1* expression in G of the VWR group in comparison to the FTR group reached statistical significance (Figure 3). In addition, in the VWR group, expression of the *Actn3* gene was downregulated in G, but strongly upregulated in S, whereas there were no major changes in the FTR group. When directly comparing the effects of the two training regimens, both of these effects reached statistical significance (Figure 3).

#### 3.3.2 Mitochondrial and metabolism‐associated genes

**Figure 4:**
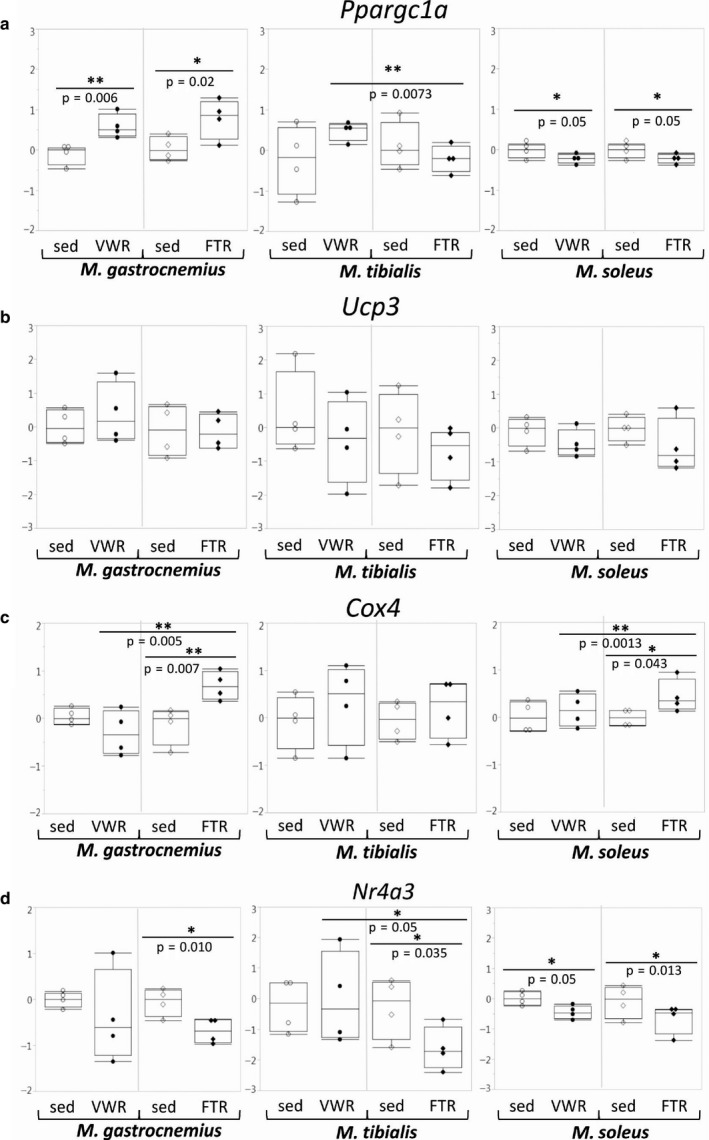
Expression of genes encoding metabolic and mitochondrial markers. Expression of the genes encoding Pgc1α, Ucp3, Cox4, and Nr4a3 was assessed by qPCR as indicated.

We found a significantly elevated expression of the *Ppargc1α* gene in G of exercised mice of both groups. In contrast, in S, the expression of this gene was significantly downregulated by both training regimens. With regard to these two muscle types, there was no significant difference when both training regimens were directly compared. In contrast, in T, there were diverging trends, resulting in a significant difference (Figure 4a). With regard to *Ucp3* expression, there were no significant effects, despite the fact that there appeared to be a general trend toward reduced expression with training (except in G/VWR) (Figure 4b). With regard to *Cox4* expression, there was a general trend toward upregulation (again except in G/VWR), which reached significance in G/FTR as well as in S/FTR. In the former, there was also a significant difference between the two training regimens (Figure 4c). For the *Nr4a3* (nuclear receptor subfamily 4 group A member 3) gene, which encodes a highly sensitive marker of skeletal muscle oxidative adaptation, we found decreased expression under all conditions, which was significant in most cases (Figure 4d).

#### 3.3.3 Inflammation‐ and anti‐inflammation‐associated genes

**Figure 5:**
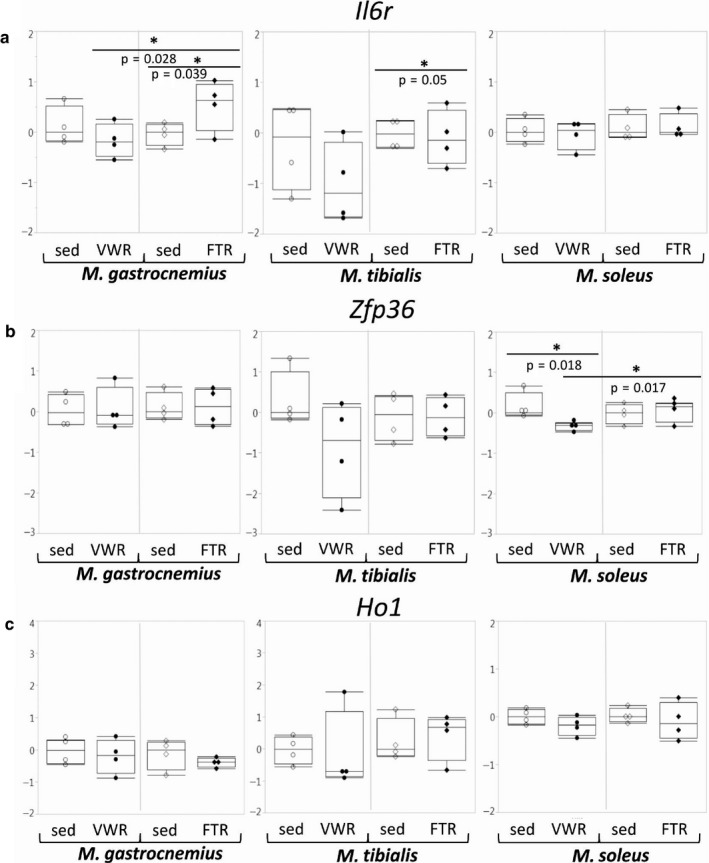
Expression of genes related to inflammation and anti‐inflammation. Expression of the genes encoding Il6r, Zfp36, and Ho1 was assessed by qPCR as indicated.

Since controlled inflammation and anti‐inflammation appear to play a major role in skeletal muscle training adaptation, we analyzed the expression of the *Il6r*, *Zfp36,* and *Ho1* genes in both groups of mice. However, we could only detect a moderate induction of *Il6r* expression in G (also significant when the two training regimens were compared), and a slight reduction in T of the FTR group (Figure 5a). In addition, we observed reduced *Zfp36* expression in S/VWR, an effect that was also significant when the two training regimens were compared (Figure 5b).

#### 3.3.4 Myostatin, Fbox32, and Murf1

**Figure 6:**
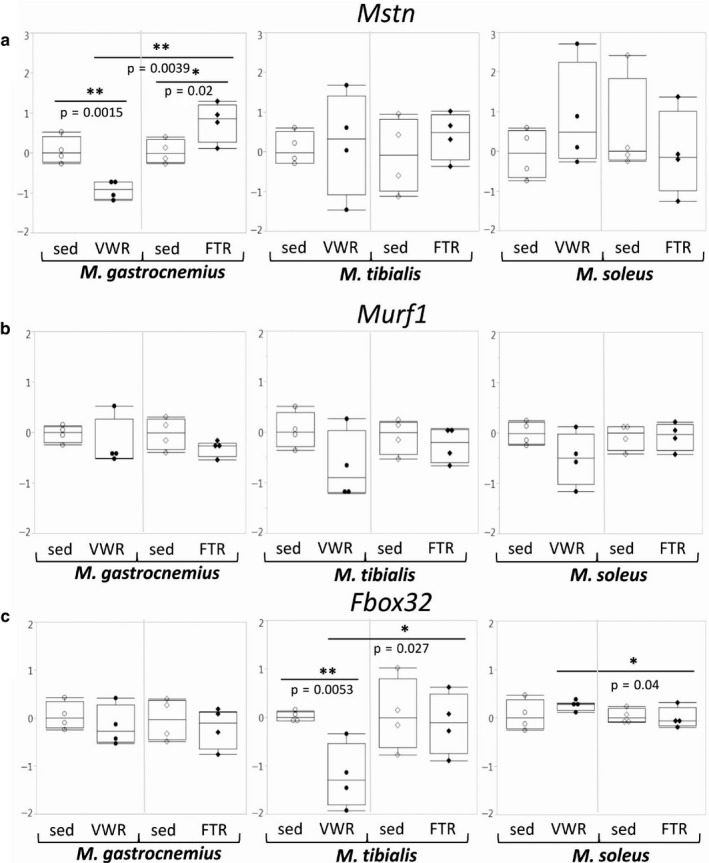
Expression of the *Mstn, Murf1,* and *Fbox32* genes. Expression of the genes encoding Mstn, Murf1, and Fbox32 was assessed by qPCR as indicated.

Expression of the *Mstn* gene was only significantly regulated in G: We observed strong downregulation in the VWR group, but moderate upregulation in the FTR group. Consequently, in this muscle, a direct comparison of the two training regimens reached (high) statistical significance (Figure 6a). In contrast, there were no significant effects on *Murf1* expression with both training regimens, although there was a trend toward reduced expression with exercise (Figure 6b). There was also a trend toward reduced expression of the *Fbox32* gene under most conditions, reaching statistical significance in T of the VWR group in comparison with the sed group and also when the effects of both training regimens were compared (Figure 6c).

#### 3.3.5 miRNAs and genes associated with the miRNA pathway

**Figure 7:**
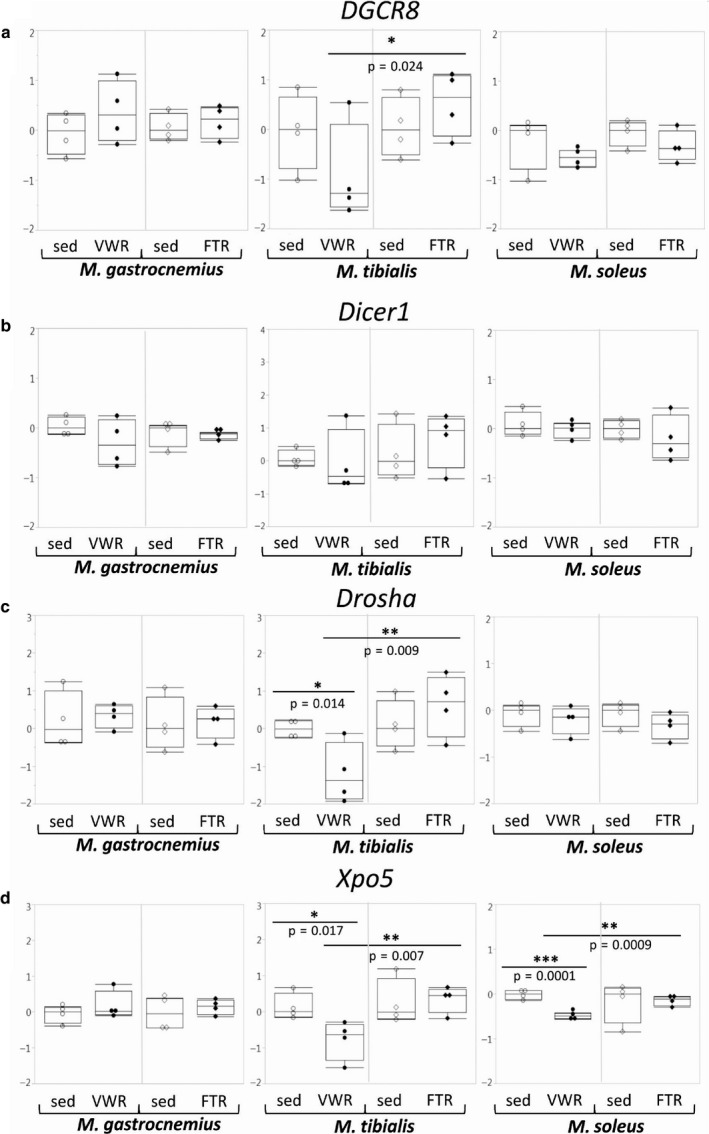
Expression of genes encoding components of the miRNA pathway. Expression of the *Dgcr8, Dicer1, Drosha,* and *Xpo5* genes was analyzed by qPCR as indicated

**Figure 8:**
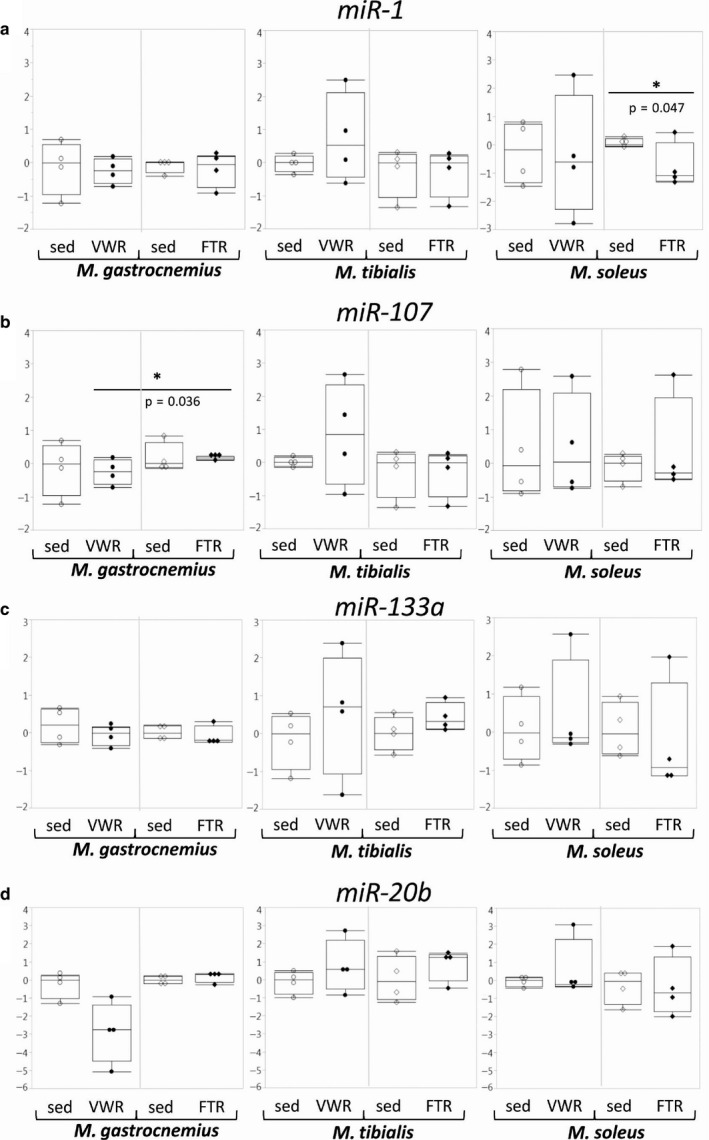
Concentrations of different miRNAs in skeletal muscle tissue. Concentrations of individual miRNAs were determined by qPCR as indicated.

Expression of genes encoding components of the miRNA pathway, such as *Drosha*, *DGCR8*, *Dicer1*, and *Xpo5*, was not significantly altered in the FTR group. In the VWR group, there was a significant downregulation of *Dicer1* in T and of *Xpo5* in both T and S (Figure 7). Overall, effects on genes encoding components of the miRNA processing machinery were minor. Furthermore, we did not find major differences with regard to concentrations of a broad variety of individual miRNAs species that we analyzed based on literature data (Kirby and McCarthy, 2013; Meurer et al., 2016; Russell and Lamon, 2015; Silva et al., 2017; Silva et al., 2020; Sjögren et al., 2018; Ultimo et al., 2018; Widmann et al., 2019; cf. Table 2) (Figure 8 and data not shown). In addition, despite the fact that there was a strong trend toward lower levels of miR‐20b in G of the VWR group (Figure 8), effects on miRNA patterns were subtle and mostly specific to individual muscles.

## 4 DISCUSSION

Our data show that both FTR and VWR had characteristic effects on skeletal muscle gene expression patterns. While some effects were similar, others differed between the two types of training. These data suggest that the two exercise programs have unique and characteristic effects on hindlimb skeletal muscle tissue.

One explanation might be that total running distances (ca. 8 km daily vs. ca. 800 m three times a week) and consequently resulting energy expenditures were probably profoundly different for the two groups of mice. Since weight gain was not significantly different, it is likely that food consumption was higher in the VWR group, which might also have affected the results. This assumption is supported by recent data by Kim et al., 2020, who also compared the effects of FTR and VWR: The authors found increased food intake in exercising, particularly in VWR, mice. Interestingly, however, whereas Kim et al. reported decreased gains in body weight in exercising, particularly in VWR, mice, in our study, weight gain in FTR (Schmitt et al., 2018) and VWR (this study) mice was not different from that observed in sedentary controls. This might be due to the fact that in the study by Kim et al., 2020, FTR mice exercised 5d/week (as compared to 3d/week in our study), and that for unknown reasons, average daily VWR running distances, as reported by Kim et al., were much higher (around 24 km/d, as compared to approximately 8 km/d in our study) (see below). In addition, in our protocol, the total duration of the training period was longer (10 weeks vs. 8 weeks for both FTR and VWR). Furthermore, our experiment does not allow control for spontaneous activity besides wheel or treadmill running, which might also have influenced the results. Isocaloric protocols would require similar food intake and complete control of activity, which is difficult to realize in accordance with animal welfare and protection regulations. Finally, whereas FTR mice were encouraged by supervisors to keep up with the moderate speed of the treadmill, that is, to run at a more or less constant speed, VWR mice appeared to run much shorter distances at a time, at a higher speed, which has also been described by others before (Manzanares et al., 2018). This running behavior, which is probably very close to natural rodent activity patterns, rather resembles a sprint than moderate‐intensity endurance training. It would be interesting to determine how both protocols affect cardiovascular fitness, specifically gains in maximum relative oxygen uptake (VO_2_max). While due to animal welfare and protection regulations, it was not possible to run mice to exhaustion, we recently analyzed the effects at least of FTR on resting heart frequency (RHF) in a similar cohort of mice, demonstrating a 10% reduction, suggesting distinct effects of this training regimen on cardiovascular adaptation (Röchner et al., submitted).

Surprisingly, we made the observation that the position of the respective cage within the cage rack affected the running behavior of some mice: When cages were positioned on a lower shelf, mice tended to run longer daily distances. Since most of the running activity occurred during the dark phase, it is unlikely that this effect is directly due to the fact that mice are exposed to higher light intensities on the upper shelves. Rather, this might be an indirect effect: Mice in the “lower” shelves might have had more rest during the day and thus be more active at night. In general, VWR activity data reported in the literature vary significantly between different studies: Whereas Kim et al., 2020, reported much higher daily running distances when compared to this study, Lightfoot et al., 2004, who analyzed daily running distances in male and female mice of different strains, found values as low as 3 km/d for male C57BL/6 mice, suggesting that a lot of yet unknown factors, such as cage position, might significantly influence running behavior.

Consistent with recent data by Kim et al., 2020, we found a trend toward increased weight of G in response to VWR. However, inconsistently, this tendency was also (even more strongly) observed in T, a muscle where Kim et al. saw no effect. In general, the observed trends toward muscle hypertrophy might be due to the fact that, as mentioned above, especially VWR is not a pure endurance, but rather sprint training in mice. In addition, different degrees of hypertrophy in individual muscles might reflect different workloads experienced by these muscles associated with running or might be due to intrinsic characteristics, such as regulatory patterns of signal transduction and transcriptional regulation. This might also explain our finding that most effects on gene regulation were strongly dependent on the type of muscle analyzed. Against this background, in the future, it will be interesting to characterize the activity and load of different muscle types during both FTR and VWR. The results would help to better understand which factors determine specific adaptation reactions of a certain muscle.

An interesting finding was the overall tendency toward higher expression levels of all *Myh* genes in G of the VWR group. This effect might reflect a general anabolic situation and be related to the trend toward muscle hypertrophy and increased weight of individual hindlimb muscles observed in this group. Correspondingly, we also observed decreased expression levels of *Mstn* in G and of *Fbox32* in T of these animals and also trends toward reduced expression of the *Murf1* gene in all muscles. Since these effects were not observed or much weaker in the FTR group, it is very likely that in contrast to FTR, VWR, which rather corresponds to an interval sprint and not a mere endurance training, has the potential to stimulate muscle growth and anabolism.

It is still not completely clear how different modes of exercise, such as FTR and VWR, influence skeletal muscle fiber type composition. Our data suggest differential expression of the “fast” *Myh1* gene and of the “intermediate” *Myh2* gene, as well as of *Actn3*, which is also a marker of “fast” fibers, in certain muscles, specifically in the VWR group, and a certain degree of fiber type switching, specifically an increased proportion of MyHC2X (*Myh1*) fibers in S/VWR. These data suggest at least a certain degree of fiber type adaptation. This was also observed in FTR mice, despite a qualitatively distinct pattern with higher proportions of type 2A fibers (Röchner et al., submitted). Reasons for the discrepancy between gene expression and fiber type staining, which was also observed in FTR mice (Röchner et al., submitted), might be that (1) mRNA versus protein/enzyme activity was assessed, that (2) fiber type staining only considers fiber proportions/numbers, but not their size/cross‐sectional area—this might specifically be important against the background that we observed a certain degree of fiber hypertrophy in VWR mice, and that (3) training might also have affected the proportion of “mixed‐type” or hybrid fibers, which cannot be quantified by means of mATPase staining. Interestingly, Kim et al., 2020, did not observe effects on fiber type specification, despite the fact that their running protocols, as mentioned above, were characterized by a higher intensity when compared to the ones employed in this study. However, their methodological approach (quantification of two troponin I isoforms by Western blot) cannot be directly compared to ours.

In addition, we found effects on metabolic markers, specifically a strong upregulation of *Ppargc1α*, encoding Pgc‐1α in G by both FTR and VWR, and upregulation of *Cox4* by FTR in both G and S. One major player in this context might be AMP‐activated kinase (AMPK), which senses energy depletion and primarily activates genes associated with oxidative metabolic pathways (for review, see Janzen et al., 2018).

The metabolic marker *Nr4a3* was downregulated in S, TA, and G. This might be due to the fact that despite the finding that this gene is strongly induced in response to acute exercise, regular training appears to exert no or even a slight inhibitory effect on its expression (for review, see Pillon et al., 2020, and references therein). This hypothesis is supported by the fact that a single bout of moderate‐intensity FTR, comparable with one training session within the regimen described here, led to a strong induction of *Nr4a3* expression in both G (6.9‐fold) and T (3.9‐fold) in our hands (Schmitt et al., 2020).

Interestingly, FTR led to a modest upregulation of the *Il6r* gene in G. This is consistent with published results obtained with human subjects and might be a means to sensitize skeletal muscle to oscillating IL‐6 levels with training (Keller et al., 2005).

In both groups, we found minor effects on genes encoding components of the miRNA processing machinery, but some distinct changes in miRNA profiles. A particularly interesting finding might be the strong trend toward downregulation of miR‐20b in G of the VWR group: Since this miRNA negatively regulates VEGF production (Lei et al., 2009), its repression might enhance angiogenesis in exercise adaptation.

In summary, these data suggest that FTR and VWR exert similar, but also differential effects on skeletal muscle, and that those adaptation properties of different muscles to different exercise regimens might be quite unique. Future, confirmatory studies should be carried out with larger animal numbers. They should also aim at directly controlling for energy expenditure and/or at analyzing the effects of a combination of both training regimens. Furthermore, a better characterization of the adaptation reactions of individual muscles might be crucial. Finally, miRNA patterns should be analyzed at a broader range and potentially also in the circulation.

## CONFLICT OF INTEREST

The authors declare no conflict of interest.

## AUTHOR CONTRIBUTIONS

Conceptualization: A.S. and B.M.; Methodology: A.S., P.H., F.R., A.‐L.B., A.F.; Writing: A.S. and B.M.; Validation: A.S., P.H., F.R., A.‐L.B., A.F.; Formal analysis: P.H. and A.S.; Visualization: P.H. and A.S.; Supervision: B.M.; Project administration: B.M.. All authors read and approved the manuscript.

## ETHICAL STATEMENT

This work does not involve human subjects. All animal experiments were approved by the local authorities and carried out under the consideration of the German animal protection law (Regierungspräsidium Tübingen, M9/14 and 35/9185.82–2).

## Data Availability

The authors confirm that the data supporting the findings of this study, except those in the context of further miRNA analyses, are available within the article. These and also raw and primary data are available from the authors upon reasonable request.
